# Regionalization of the SWAT+ model for projecting climate change impacts on sediment yield: An application in the Nile basin

**DOI:** 10.1016/j.ejrh.2022.101152

**Published:** 2022-08

**Authors:** Albert Nkwasa, Celray James Chawanda, Ann van Griensven

**Affiliations:** a Hydrology and Hydraulic Engineering Department, Vrije Universiteit Brussel (VUB), 1050 Brussel, Belgium; b Water Science & Engineering Department, IHE Delft Institute for Water Education, 2611 AX Delft, the Netherlands

**Keywords:** Soil erosion, Sediment yield, SWAT+, Regional modeling, Climate change, Nile basin

## Abstract

**Study region:**

Nile basin.

**Study focus:**

Several studies have shown a relationship between climate change and changes in sediment yield. However, there are limited modeling applications that study this relationship at regional scales mainly due to data availability and computational cost. This study proposes a methodological framework using the SWAT+ model to predict and project sediment yield at a regional scale in data-scarce regions using global datasets. We implement a framework that (a) incorporates topographic factors from high/medium resolution DEMs (b) incorporates crop phenology data (c) introduces an areal threshold to linearize sediment yield in large model units and (d) apply a hydrological mass balance calibration. We test this methodology in the Nile Basin using a model application with (revised) and without (default) the framework under historical and future climate projections.

**New hydrological insights for the region:**

Results show improved sediment yield estimates in the revised model, both in absolute values and spatial distribution when compared to measured and reported estimates. The contemporary long term (1989 – 2019) annual mean sediment yield in the revised model was 1.79 t ha^−1^ yr^−1^ and projected to increase by 61 % (44 % more than the default estimates) in the future period (2071 – 2100), with the greatest sediment yield increase in the eastern part of the basin. Thus, the proposed framework improves and influences modeled and predicted sediment yield respectively.

## Highlights

• Proposed framework to improve regional SWAT+ sediment yield estimates under climate projections.
• Framework tested in a data-scare region (Nile basin) with existing global datasets.
• Results show improved contemporary sediment yield estimates both in absolute values and spatial distribution.
• Long-term mean annual sediment yield in the Nile basin is projected to increase by 61 % in the future period (2071 – 2100).

## Graphical Abstract

**Figure:**
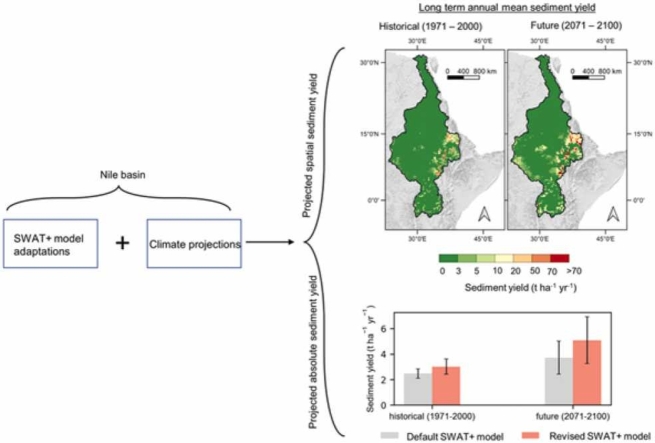

## 1 Introduction

With the need to keep soil erosion within the focus of all major environmental and agricultural policies such as the Sustainable Development Goals (SDGs) and soil and nature conservation, large scale sediment yield modeling and mapping are required (Tamene and Le, 2015, Alewell et al., 2019). Moreover, regional to continental sediment yield datasets bridge the knowledge gaps in water quality, food security, hydrology and climate change mitigation scenarios (Zhu et al., 2010, Alewell et al., 2019). However, measuring sediment yield across the whole landscape is impractical. Thus, computational modeling is often applied to estimate soil loss, upon which mitigation and global change scenarios may be based (Bosco et al., 2015). Despite the efforts, assessing sediment yield at large spatial scales remains one of the main challenges in sediment yield modeling, not the least due to high computational demands, especially for process-based models (Eekhout et al., 2018). As a compromise between accuracy and practicality, models often utilize input data of low spatial resolution, restrict the number of simulated model units while adopting simplifications that ignore physical landscape processes (Vigiak et al., 2015, Nkwasa et al., 2022). Subsequently, research is continuously needed to improve modeling approaches for estimating sediment yield at large spatial scales.

The Soil and Water Assessment Tool (SWAT; Arnold et al., 1998) is among the most well-known semi-distributed models that simulate sediment yield and river sediment transport using hydrological and sediment modules. SWAT utilizes the Modified Universal Soil Loss Equation (MUSLE; Williams, 1975) to predict sediment yield at Hydrologic Response Unit (HRU) level. The model assumes that all the eroded sediments within the HRU reach the river (Lenhart et al., 2005, de Vente et al., 2013). SWAT has been widely used for sediment yield modeling in several river basins across the globe at different spatial scales (Betrie et al., 2011, Tibebe and Bewket, 2011; Mosbahi et al., 2013; Phuong et al., 2014; Bonumá et al., 2015; Vigiak et al., 2017; Aga et al., 2018; Markhi et al., 2019; Mosbahi and Benabdallah, 2020; Shrestha et al., 2020) with limited applications on regional to continental sediment yield modeling. Previous studies have highlighted the sediment yield sensitivity issues of the model with regards to the resolution of input data (Cotter et al., 2003, Luzio et al., 2005, Lin et al., 2013) and the spatial scale dependency of the MUSLE (Chen and Mackay, 2004, Vigiak et al., 2015, Gwapedza et al., 2018a).

Among the input data, Cotter et al. (2003) shows that the SWAT model output is most sensitive to the digital elevation model (DEM). Unlike streamflow, which is largely unaffected by DEM pixel size (Malagò et al., 2018, Tan et al., 2018), sediment yield estimates decrease significantly with the increasing cell size of the DEM, as the cell aggregation reduces the spatial variability (Wu et al., 2005, Chaplot, 2014, Sharma and Tiwari, 2014, Li et al., 2021). Cell aggregation significantly influences the slope steepness and slope length topographic factors (Lin et al., 2013, Mukherjee et al., 2013). Topographic factors have the most significant influence on sediment yield among the MUSLE factors in several basins (Kruk et al., 2020). Hence, large scale SWAT applications that often utilize coarser resolution DEMs substantially underestimate the sediment yield simulations. One common alternative that has not been investigated in large scale SWAT applications is the use of topographic factors (slope data) extracted from medium/high-resolution DEMs and resampled to the coarser resolution of the model setup. Grohmann (2015) recommended using resampled morphometric parameters (slope and aspect) derived from a higher resolution DEM than morphometric parameters derived from a resampled DEM.

Additionally, Chen and Mackay (2004) highlighted the nonlinear relationship of the MUSLE between the HRU sediment yield and the HRU area in the SWAT model, affecting the conservation of sediment generation among different levels of watershed partitioning. However, Chiang and Yuan (2015) argue that the runoff concentration times increase as the HRU area increases, resulting in lower sediment yield estimates which neutralizes the nonlinear relationship between sediment generation and HRU area. Other studies (e.g. Betrie et al., 2011; de Vente et al., 2013; Duru et al., 2018; Dutta and Sen, 2018; Markhi et al., 2019) have also shown that calibrating the MUSLE parameters can indirectly correct for the overestimations or underestimations of the sediment yields. However, matching the sediment loadings at the watershed outlet does not mean that the internal processes are correctly represented (Vigiak et al., 2015, Gwapedza et al., 2021). Gwapedza et al. (2018a) recommends that efforts be made by users of the MUSLE to minimize the spatial scale effects as far as possible. For example; Vigiak et al. (2015) proposed an approach that introduces a threshold area in the MUSLE above which sediment yields are linearized in relation to the HRU area. By setting the threshold area at 0.01 km^2^ for a 132,000 km^2^ catchment, Vigiak et al. (2015) showed that the modification significantly impacted sediment yields at both hillslope and basin scale. On the other hand, increasing the threshold area may conceptually account for other sediment sources and sinks (de Vente and Poesen, 2005). Within the Danube basin, Vigiak et al. (2017) set a threshold of 50 km^2^ which was consistent with the MUSLE range of application. The potential effect of this approach in minimizing the spatial scale dependency of the MUSLE is further explored in this study.

According to Morgan and Nearing (2011) and Zhao et al. (2013), the cover management factor is probably among the different sediment yield factors, the most important with crop cover playing an important role in controlling soil erosion (Gyssels et al., 2005, Bosco et al., 2015; Panagos et al., 2015). However, simplified approaches in representing agricultural land use are usually adopted at a large scale in regional hydrological models (Srivastava et al., 2020, Nkwasa et al., 2022). Previous applications of the SWAT model (Schuol and Abbaspour, 2006, Schuol et al., 2008) and SWAT+ model (Chawanda et al., 2020) at a regional scale in Africa have utilized the default generic way of representing agricultural land use without management practices affecting the parametrization of the cover management factor for cultivated areas and subsequent sediment yields. However, Nkwasa et al. (2022) suggested a methodological approach that utilizes global crop phenology datastes and decision tables to incorporate crop phenology and the associated management practices of planting, harvesting, irrigation and fertilization in regional SWAT+ model applications. This approach, coupled with the MUSLE equation, could improve crop cover estimation for cultivated areas by accounting for the correct temporal variation.

Several sediment yield studies (e.g. Tibebe and Bewket, 2011; da Silva et al., 2013; Mosbahi et al., 2013) have shown a strong correlation between surface runoff and soil loss. The MUSLE explicitly considers surface runoff in estimating the sediment yield. Hence, an accurate simulation of surface runoff is essential. The SWAT model was originally intended for use in large ungauged watersheds (Arnold et al., 1998, Srinivasan et al., 2010). In many studies where observed data is available, calibration is done using a time series of flow data at the outlet to improve the model performance. However, this does not mean that the internal processes such as surface runoff are correctly represented. Chawanda et al. (2020) proposed a hydrological mass balance calibration (HMBC) approach to calibrate large scale regional models. HMBC utilizes soft data (information on individual processes e.g. surface runoff, evapotranspiration and groundwater) to enhance the calibration process for large scale models at lower computation and time costs.

This paper proposes a methodological framework using the SWAT+ model (Bieger et al., 2017, Arnold et al., 2018) to improve regional scale predictions of sediment yield in a data scare region and where relatively coarse spatial data is utilized. More specifically, the framework involves; (1) assessing and adopting an approach to utilize topographic factors extracted from medium/fine resolution DEMs and resampled to a coarser SWAT+ model resolution, (2) incorporating and assessing the approach proposed by Vigiak et al. (2015) for reducing the sensitivity of sediment yields to the HRU area in the MUSLE, (3) adopting the approach proposed by Nkwasa et al. (2022) for incorporating crop phenology and agricultural management in regional SWAT+ model applications with respect to sediment yield estimates, (4) applying the regional hydrological mass balance calibration approach (Chawanda et al., 2020) to improve model surface runoff prediction. We compare the influence of the methodological framework on sediment yield estimates and river sediment loads against measured and reported local estimates in the region.

We further evaluate the influence of the proposed methodological framework on sediment yield projections under climate change using a scenario analysis involving the Shared Socioeconomic Pathway and Representative Concentration Pathway (SSP5-RCP8.5 – high CO_2_ emission scenario) to demonstrate how the proposed framework can impact projected sediment yield estimates under climate change. This research provides an overall representation of the Nile basin’s current and future sediment yield rates under climate change and may be used by policy makers as input in decision-making to design suitable practices for adapting and mitigating climate change impacts on sediment yield. In addition, the modeling framework proposed here can be adopted in other regional studies to estimate sediment yield under climate change.

## 2 Materials and methods

### 2.1 Study Area

**Fig. 1:**
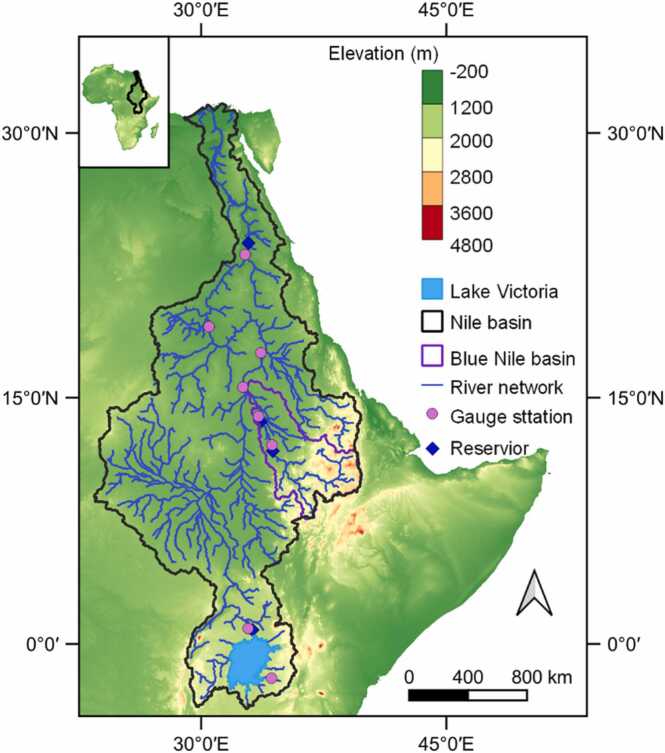
Study area – Nile basin.

Our study area in the Northeast part of Africa (Fig. 1) is about 3489,000 km^2^. This area covers the Nile basin countries including Kenya, Tanzania, Uganda, Burundi, Rwanda, Ethiopia, Egypt, Sudan and South Sudan. The region's climate is characterized by a strong latitudinal wetness gradient and a spatially contrasted mean annual rainfall distribution (Camberlin, 2009). The mean annual rainfall varies from 1000 to 1500 mm, falling to almost zero in the lower parts of the basin (Kirby et al., 2010). The Nile basin has several sub-basins such as the Blue Nile, Victoria Nile, White Nile, Atbara, Baro-Akobo-Sobat, Bahr El jebel and Bahr El Ghazal. The Blue Nile contributes about 60 % of the total flow of the Nile while Atbara and Baro-Akobo-Sobat contribute slightly less than 15 % each (Beyene et al., 2010). The geology and structural setup of the basin is characterized by crystalline basement rocks ranging in age from Precambrian to Quaternary, which determines its hydrogeomorphic nature (Garzanti et al., 2006). However, the uplands (mainly in Ethiopia), are occupied by volcanic rocks (Alemayehu et al., 2017, Alemayehu et al., 2017).

Several studies in the region have highlighted high sediment yield estimates which have been attributed mainly to topography, rainfall and land conversion to agriculture (Vanmaercke et al., 2014, Blake et al., 2018). However, most of the studies (e.g. Fenta et al., 2016; Haregeweyn et al., 2017; Ebabu et al., 2019; Yibeltal et al., 2019) have focused on assessing soil erosion and sediment yield at plot to watershed scales. Fenta et al. (2020) used the RUSLE method to estimate water and wind erosion across East Africa, which represents a great advancement in mapping large scale soil loss estimates within the region. Nevertheless, with limited regional studies, the geographical distribution of sediment yield remains roughly known. Accordingly, the lack of sufficient information on regional sediment yields poses significant challenges to national and regional planning towards soil erosion in the region (Fenta et al., 2020).

### 2.2 SWAT+ model

SWAT+ model (Arnold et al., 2018) is a completely restructured version of the SWAT (Arnold et al., 1998) model, which offers greater flexibility with connecting spatial units in the representation of management operations (Bieger et al., 2017). In SWAT+ , a basin is divided into sub-basins connected by a stream network, further divided into Land Scape Units (LSUs) and HRUs. LSUs allow the separation of upland processes from wetlands (Bieger et al., 2017), while HRUs are areas of unique properties of land use, soil, slope class and management practices (Neitsch et al., 2005). The HRU discretization was preferred in this study due to the resonable computational demands at a regional scale, compared to a grid discretization that requires significant computational requirements (Pignotti et al., 2017). Simulations in the model are done at two phases; a land phase – at the HRU level and a stream phase – at the reach (sub-basin) level (Neitsch et al., 2005). The land phase deals with surface runoff, sediment yield, evapotranspiration (ET), nutrient and soil-water process simulations. In contrast, the routing phase defines the water, sediment and nutrient movement through the channel network to the outlet (Bouslihim, 2020). SWAT+ has not been widely used since it is relatively new, unlike SWAT that has been applied across the world in various sediment yield studies (Betrie et al., 2011, Tibebe and Bewket, 2011, Vigiak et al., 2017, Dutta and Sen, 2018, Mosbahi and Benabdallah, 2020, Shrestha et al., 2020).

#### 2.2.1 Sediment yield generation

The SWAT+ model estimates sediment yield using the MUSLE for each HRU (Neitsch et al., 2005). Compared to the USLE (Universal Soil Loss Equation), the MUSLE (Eq. 1) uses the surface runoff and peak flow rate, which increases the model prediction accuracy since there is no need for a delivery ratio and the sediment yields of single storms can be estimated (Shen et al., 2009). The MUSLE estimates sediment yields not gross erosion (Vigiak et al., 2015).

(1) $$\mathrm{SY}=11.8\;{\left(Q_{\mathrm{surf}}q_{\mathrm{peak}}A_{h\mathrm{ru}}\right)}^{0.56}\;x\;K_{\mathrm{USLE}}\;x\;C_{\mathrm{USLE}}\;x\;P_{\mathrm{USLE}}x\;{\mathrm{LS}}_{\mathrm{USLE}}\;x\;\mathrm{CFRG}\;$$

Where; SY is the sediment yield (tons/day), $Q_{\mathrm{surf}}$ is the surface runoff volume (mm day^−1^), $q_{\mathrm{peak}}$ is the peak runoff rate (m^3^ s^−1^), $A_{\mathrm{hru}}$ is the area of the HRU (ha), $K_{\mathrm{USLE}}$ is the USLE soil erodibility factor, $C_{\mathrm{USLE}}$ is the USLE crop management factor, $P_{\mathrm{USLE}}$ is the USLE support practice factor, ${\mathrm{LS}}_{\mathrm{USLE}}$ is the USLE topographic factor and CFRG is the coarse fragment factor.

However, Vigiak et al. (2015) modified Eq. (1) to Eq. (2) to reduce the impact of the HRU area on the estimation of the sediment yield (Chen and Mackay, 2004), by introducing a threshold area ($A_{t}$) above which sediment yields are linearized proportionally to the HRU area. However, this approach holds for non-urban HRUs. For urban HRUs, the buildup/wash-off approach applied by default is retained (Neitsch et al., 2005).

(2) $$\mathrm{SY}=[11.8{\left(Q_{\mathrm{surf}}{q_{r}A}_{m}\right)}^{0.56}x\;K_{\mathrm{USLE}}\;x\;C_{\mathrm{USLE}}\;x\;P_{\mathrm{USLE}}x\;{\mathrm{LS}}_{\mathrm{USLE}}\;x\;\mathrm{CFRG}](\frac{A_{\mathrm{hru}}}{A_{m}})$$

Where; $q_{r}$is the peak runoff rate (m^3^/s) linearized by area (Eq. 3)

(3) $$q_{r}=\;q_{\mathrm{peak}}\left(\frac{A_{m}}{A_{\mathrm{hru}}}\right);A_{m}=\min(A_{\mathrm{hru}},\;A_{t})$$

Where; $A_{m}$ is the minimum area between the HRU area ($A_{\mathrm{hru}}$) and the threshold area ${(A}_{t}$) which can be conceptualized as the largest hydrologically isolated unit occurring within a large HRU.

### 2.3 Datasets and default model setup

Global datasets used in this research were obtained from freely available data. The datasets included; the Digital Elevation Model (DEM) downloaded from Shutter Radar Topography Mission (SRTM; Farr et al., 2007) at 90 m resolution, the Harmonized land use (LUH2; Hurtt et al., 2020) at 0.25^0^ resolution, soil map at 250 m resolution from Africa Soil Information Service (AFSIS; Hengl et al., 2015), Weather inputs at a 0.5^0^ resolution from the global observational dataset GSWP3-W5E5, which is a merge between the GSWP3 (Global Soil Wetness Projected phase 3) data set (Dirmeyer et al., 2006, Kim et al., 2017) and the W5E5 dataset (Lange, 2019a, Cucchi et al., 2020), Crop phenology (plant and harvest dates) from Global Gridded Crop Model Intercomparison (GGCMI; Jägermeyr et al., 2021) at 0.5^0^ resolution, irrigated areas from Food and Agriculture Organization (FAO; Siebert et al., 2013) at 0.083^0^ resolution, Elemental Nitrogen and Phosphorus fertilizer from Hurtt et al. (2020) and Lu and Tian (2017) at 0.5^0^ resolution respectively, discharge data from Global Runoff Data Centre (GRDC; http://grdc.bafg.de) at a monthly timestep, reservoir data from the Global Reservoir and Dam (GRanD) database (Lehner et al., 2011) and ET at 250 m resolution from FAO Water Productivity Open-access portal (WaPOR; https://wapor.apps.fao.org).

The default SWAT+ model (revision 60.5) was set up with the QGIS from 1950 to 2019. The harmonized land use product is formatted as NetCDF; thus, the SWAT+ code was adapted to include subroutines to read the NetCDF data using an approach proposed by Chawanda et al. (2020). The study area was discretized into 768 landscape units and 63,622 HRUs based on soil, land use and slope. The number of HRUs was selected to balance the computational cost while reducing the error due to lumping effects. The discharge data from GRDC was manually reviewed and checked for inconsistencies. Only stations with four or more years of data above 1950 were retained in the calibration process.

### 2.4 Model adaptations and parameterization

#### 2.4.1 Topographic factor

The topographic factor, ${\mathrm{LS}}_{\mathrm{USLE}}$ (Eq. 4) reflects the effect of surface topography (combination of slope steepness and slope length) on sediment yield. The SWAT+ model derives its topographic factors (slope steepness and slope length) from the DEM at the HRU level. The model sets the slope length based on a lookup table that relates the slope length to the HRU slope gradient (Malagò et al., 2018). It is worth noting that a resampled DEM (90–250 m) was used during the default regional model setup to overcome the high computational demands during setup. Hence, the default model's topographic factors (slope steepness and slope length) were extracted from this resampled DEM (250 m).

(4) $${\mathrm{LS}}_{\mathrm{USLE}}=({\frac{L}{22.1})}^{m}\;x\;[65.41\;x\;\sin^{2}\left(\propto \right)+4.56\;x\;\sin(\propto )\;+0.065]$$

Where:$\;{\mathrm{LS}}_{\mathrm{USLE}}$ is the topographic factor, L is the slope length, m is the exponential term, and ∝ is the angle of the slope. The exponential term, m is calculated as in Eq. (5).

(5) $$m=0.6\;x\;(1-\exp\left[-35.835\;x\tan\left(\propto \right)\right])$$

**Fig. 2:**
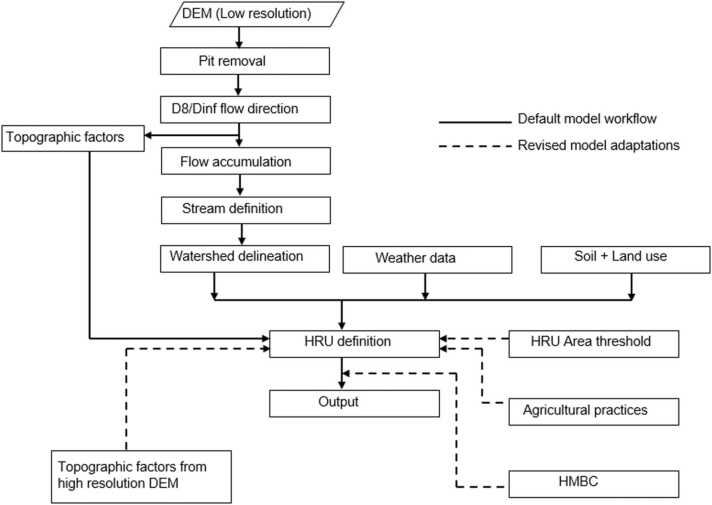
Methodological adaptations in the SWAT+ sediment yield modeling framework.

To overcome the impact of the DEM derivatives (topographic factors) on the model outputs, specifically sediment yield, an alternative approach that uses topographic factors extracted from a medium resolution DEM (90 m) and resampled to the coarser resolution of the model was adopted (Fig. 2). These topographic factors were extracted from the DEM using the same algorithm used by SWAT+ to generate flow direction and slope, i.e. Terrain Analysis Using Digital Elevation Models (TauDEM; https://hydrology.usu.edu/taudem/taudem5/index.html).

The use of a 90 m DEM ensured the computation of the topographic factors maintaining a scale congruent with the one used during the USLE’s experimental plots (Borrelli et al., 2017). This approach was first tested in the Blue Nile basin (Fig. 1) using topographic factors derived from resampled DEMs (250 m, 500 m and 1000 m resampled from 90 m) and resampled topographic factors derived from a 90 m DEM (Supporting material A). The approach was later extended to the regional Nile basin model. The default model topographic factors originally extracted from a resampled 250 m DEM were replaced at the HRU level by resampled topographic factors extracted from the 90 m DEM.

#### 2.4.2 HRU area threshold

The approach proposed by Vigiak et al. (2015) in Eq. (2) and Eq. (3), to reduce the impact of HRU area on the estimation of the sediment yield by applying a threshold area was adopted in this study (Fig. 2). The relationship between a peak in the sediment yields and HRU area was used as a rule to select the threshold area ($A_{t}$). The same concept was used by Vigiak et al. (2017) within the Danube basin. Considering that the MUSLE was developed to assess sediment yields for catchments of area up to 40 km^2^, HRUs were thus conceptually interpreted as small catchments. The threshold area was defined as the area at which the relationship between sediment yields and catchment size would peak. Using the compiled sediment yield data from previous local studies in Africa (Vanmaercke et al., 2014), 41 catchment sizes of up to 625 km^2^ comparable to the largest model HRU size were used to guide the threshold area selection.

#### 2.4.3 Crop land use and cover factor

The SWAT+ model simulates agricultural land use by default in a generic way where the phenological development of crops from planting is driven by accumulated heat units (Arnold et al., 1998). However, this approach may not work for tropical and sub-tropical regions where crop growth is mainly controlled by rainfall rather than temperature (Alemayehu et al., 2017, Alemayehu et al., 2017, Nkwasa et al., 2020). Hence, the use of heat units in crop scheduling could lead to inconsistencies in crop phenology simulations, especially for tropical and subtropical regions. In turn, affecting the estimation of the cover and management factor ($C_{\mathrm{USLE}}$) in the MUSLE for cultivated areas. As an alternative, Nkwasa et al. (2022) proposed an approach to incorporate crop phenology and associated management practices at a large scale using global datasets adopted in this study (Fig. 2). Technical details on the implementation of this approach for this case study can be found in Nkwasa et al. (2022).

SWAT+ updates the cover and management factor daily using Eq. (6) because the plant cover varies during the plant’s growth cycle (Neitsch et al., 2005).

(6) $$C_{\mathrm{USLE}}=\exp([\left(\mathrm{In}\left(0.8\right)-\mathrm{In}\left(C_{m}\right)\right)x\exp\left[-0.00115x{\mathrm{rsd}}_{s}\right]+\mathrm{In}\left[C_{m}\right])$$

Where; $C_{\mathrm{USLE}}$ is the cover management factor, $C_{m}$ is the minimum value for the cover management factor for the land cover, and ${\mathrm{rsd}}_{s}$ is the amount of soil surface residue (kg ha^−1^). $C_{m}$ can be estimated from a known average annual factor using Eq. (7) (Arnold and Williams, 1995).

(7) $$C_{m}=1.463\mathrm{In}\left[C_{a}\right]+0.1034$$

Where; $C_{a}$ is the mean annual $C_{\mathrm{USLE}}$ factor for the land cover.

#### 2.4.4 Other MUSLE parameters

Other factors influencing sediment yield were set as follows; Soil erodibility, $K_{\mathrm{USLE}}$ was estimated according to the equations described by Williams (1995) and Neitsch et al. (2011) using the AfSIS database (Hengl et al., 2015) at 250 m resolution. The coarse fragment factor, CFRG was estimated as the percentage of rock in the first soil layer using the equations described in Neitsch et al. (2011). The support practice factor, $P_{\mathrm{USLE}}$ was set according to the land use and land cover (LULC). The Wischmeier and Smith (1978) table of $P_{\mathrm{USLE}}$ was used to set the $P_{\mathrm{USLE}}$ factor values for agricultural land use depending on the support practice and the maximum slope length. A cross-slope support practice was assumed for all agricultural areas. However, a value of 1 was set for all non-agricultural land uses and land uses where no conservation was found to be practiced.

### 2.5 Sensitivity analysis

Sensitivity analysis is done to evaluate the influence of parameters on model output (Shoemaker and Benaman, 2003). The response of each MUSLE parameter was measured using a simple deterministic sensitivity analysis approach developed by (Benaman, 2002). Maximum and minimum possible values of each MUSLE factor for this region selected from literature (Kassam et al., 1991, Nill et al., 1996, Balabathina et al., 2020, Girmay et al., 2020, Kebede et al., 2021) were used in the sensitivity analysis. Initially, the model was run using the basin factor values used as baseline values. Subsequently, the model was run with the minimum and maximum factor values respectively, one at a time to evaluate the sediment yield variations in the model. According to Loucks et al. (2005) and Gwapedza et al. (2018b), using such an approach may reflect differences in model outputs between maximum and minimum values for each factor, showing the most sensitive factors.

### 2.6 Model calibration and evaluation

#### 2.6.1 Hydrological mass balance calibration (HMBC)

Arnold et al. (2015) recommended soft calibration to improve process representation by more realistically simulating the hydrological mass balance components such as the Surface run off ($Q_{\mathrm{surf}}$), that is directly used in the MUSLE. Consequently, the HMBC (Chawanda et al., 2020) approach was adopted in this study (Fig. 2) using the following general method;.

(1) The subbasins were divided into calibration zones based on expert judgement (Supporting Material B, Fig. B1). Long-term averages of water balance components for each calibration zone were derived from global datasets, i.e. global observational precipitation dataset (GSWP3-W5E5), ET from WaPOR, surface runoff extracted from the GRDC discharge data using WETSPRO (Willems, 2009) and groundwater (precipitation minus ET and surface runoff). Using the water balance output file from SWAT+ , the corresponding long-term averages of water balance components for each calibration zone were extracted.
(2) The input data for the HMBC was expressed as water balance ratios (Supporting Material B, Table B1) and selected parameters (Table 1) were adjusted automatically for each region using an algorithm written in python (Chawanda et al., 2020). The parameters were selected based on their influence on the water balance component using previous (https://swat.tamu.edu/publications/calibrationvalidation-publications/) SWAT calibration and validation literature.

**Table 1:** Selected model calibration parameters (See; Arnold et al., 2013 for details about model parameters).

Parameter	Definition	Significance
cn2	Curve number	Directly affects the surface runoff component
esco	Soil evaporation compensation factor	Directly affects the ET component
epco	Plant uptake compensation factor	Directly affects the ET component
alpha	Baseflow alpha factor	Directly affects the subsurface flow component
lat_len	Slope length for lateral subsurface flow	Directly affects the subsurface flow component

#### 2.6.2 Comparison of SWAT+ sediment yield estimates with observed and reported sediment yield estimates

Formal independent validation of sediment yields at a regional or larger scale is not feasible due to a lack of long-term field-scale measurements (Kirkby et al., 2004). Furthermore, field or plot measurements are mainly carried out in croplands or erosion-prone areas, which do not capture the landscape variability (Elnashar et al., 2021). Consequently, sediment yield evaluation followed a cross-comparison of modeling results from previous measured and reported local studies in the region. A compiled dataset of observed and reported sediment yield estimates mainly from Vanmaercke et al. (2014) was utilized in a cross-comparison of modeling results. Validating model simulations using previous estimates is not very reliable, although it provides a starting point in the context of data scarcity. Comparison was made on long-term sediment yield rates as one-to-one comparisons were prevented by the differences in the temporal and spatial scales of the previous local studies and model simulations. Further insights were obtained by calculating the spatial correlation of the model sediment yield estimates with observed and reported sediment yield estimates. Due to the limited availability of regional river sediment yield data, calibration of river sediment yield (RSSY) was not carried out. However, the simulated long term annual average river sediment loads across the river network were evaluated against observed and published estimates from literature.

The model setup with the proposed adaptations, i.e. slope adaptation (to overcome the impact of the DEM derivatives on the model outputs), HRU area threshold (to reduce the spatial scale effects of HRUs on the estimation of the sediment yield), improved agricultural land use representation (to improve crop cover estimation for cultivated areas) and HMBC (to calibrate large scale regional models), is hereafter referred to as the ‘revised model’ in the subsequent sections. These adaptations are expected to improve the robustness of the revised model in sediment yield prediction.

### 2.7 Climate change scenarios for soil loss projections

**Fig. 3:**
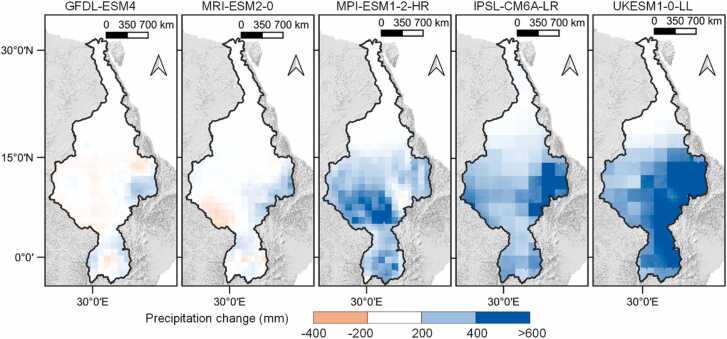
Change in annual mean precipitation from the periods 1971–2000–2070–2100 projected from five GCMs under CMIP6.

In order to explore how the two setups (default and revised model setups) affect sediment yield projections under climate change, we used 5 GCMs (GFDL-ESM4, IPSL-CM6A-LR, MPI-ESM1–2-HR, MRI-ESM2–0 and UKESM1–0-LL) from the bias-corrected CMIP6 climate forcing data (Lange, 2019b, Lange et al., 2021) for historical and future SSP5-RCP8.5 conditions. The five GCMs were selected to show the possible future precipitation pattern (Fig. 3) under the SSP-RCP8.5 scenario representing the high end of plausible future pathways. The 5 selected GCMs mostly project an increase in precipitation (Fig. 3) within the region especially in the Ethiopian highlands which represents the CMIP6 (Coupled Model Intercomparison Project) mean model ensemble under the SSP5-RCP8.5 scenario (Almazroui et al., 2020).

For the historical reference, we ran both the default and revised SWAT+ models from 1971 to 2000 and for the future scenario from 2070 to 2100. In total, we run 20 simulations with GCM forcing (i.e. 2 model setups, each forced with 5GCMs for both the historical and future scenarios).

## 3 Results and discussion

### 3.1 Sensitivity analysis

The sensitivity analysis results (Table 2) revealed that the model was more sensitive to the parameter relating to the topographic factor (${\mathrm{LS}}_{\mathrm{USLE}}$) compared to other parameters despite using a low maximum (${\mathrm{LS}}_{\mathrm{USLE}}$) factor for the region. The minimum and maximum ranges for the ${\mathrm{LS}}_{\mathrm{USLE}}$ parameter gave the lowest and highest default model outputs respectively. The model output associated with the maximum ${\mathrm{LS}}_{\mathrm{USLE}}$ were > 60 % higher than the outputs given by setting other parameters to a maximum value. A similar trend was observed with the model outputs associated with the minimum ${\mathrm{LS}}_{\mathrm{USLE}}$ that were > 63 % lower than the model outputs given by setting other parameters to minimum values.

**Table 2:** Sensitivity of sediment yield estimates to model input parameter values.

Parameters	Parameter ranges		Sediment yield (t ha^−1^ yr^−1^)	
	Minimum	Maximum	Minimum	Maximum
Cover, $C_{\mathrm{USLE}}$	0.001	0.70	1.84	7.80
Soil erodibility, $K_{\mathrm{USLE}}$	0.001	0.65	0.24	9.86
Topography, ${\mathrm{LS}}_{\mathrm{USLE}}$	0	10.0	0	24.30
Practice, $P_{\mathrm{USLE}}$	0.01	1.0	0.04	1.52

It is important to point out that some few studies such as; Girmay et al. (2020) reported a maximum ${\mathrm{LS}}_{\mathrm{USLE}}$ factor range of 100–311 occupying 1.5 % in Agewmariayam watershed, northern Ethiopia and Balabathina et al. (2020) reported a maximum ${\mathrm{LS}}_{\mathrm{USLE}}$ factor of 96.47 occupying 1.6 % of the Lake Tana basin in Ethiopia which further shows the diverse sensitivity of the topographic factor in the region. Additionally, Claessens et al. (2008) also found out that the topographic factor was the main factor defining the large potential erosion patterns in East Africa. These results underscore the importance of critically considering the topographic factor in sediment yield studies within the Nile basin region as it has a profound impact on the magnitude of soil loss.

### 3.2 Topographic factor adaptation

**Fig. 4:**
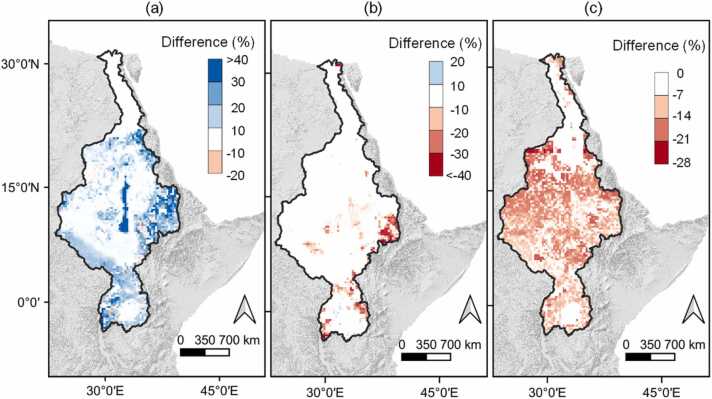
(a) Model differences after topographic factor adaptation; (b) Model differences after crop cover adaptation; (c) Model differences after HRU area adaptation.

By replacing the topographic factors extracted from the 250 m DEM resolution with resampled topographic factors extracted from a 90 m DEM resolution, the default annual average sediment yield estimates increased by 38.4 % with most of the high elevation areas having a greater percentage increase (Fig. 4(a)). This increase offsets the original underestimation of the sediment yield estimates caused by coarse slope data extracted from a resampled 250 m DEM.

Table C1, Supporting material C shows how the underestimation of sediment yield is consistently pronounced in the default model estimates which could be attributed to the coarse slope data derived from a resampled 250 m DEM. Resampling a high resolution DEM prior to calculating slope and slope length attenuates relief and slope systematically decreases as the resolution gets coarser. A reduction in slope and slope length causes a lower ${\mathrm{LS}}_{\mathrm{USLE}}$ factor, resulting into a reduction in sediment yield output. However, resampled slopes are more correlated to original ones with a smaller decrease in maximum values and similar mean values (Grohmann, 2015).

### 3.3 Crop land use representation

Fig. 4(b) shows the impact of improved agricultural representation on sediment yield estimates for cultivated areas. Sediment yield is reduced in cultivated HRUs with the improvement in agricultural land use representation because the crop canopy cover grows within the correct cropping season (mainly corresponding to the start of the rainy season), which reduces the effective energy of the intercepted raindrops. Consequently, the crop cover factor estimation is improved to correlate with the growing seasons (Supporting material D, Fig. D1), i.e. crop cover factor is lowest in the rainy seasons which implies a strong crop cover on the soil layer.

The reduction in sediment yield estimates goes over 40 % (Fig. 4(b)) in some cultivated areas, with the average regional sediment yield reduction of 7.8 %. However, the single cropping season per year provided by the global phenology datasets (Jägermeyr et al., 2021) leads to the representation of only a single cropping season even for areas with multiple cropping seasons. This means that some cultivated areas are bare in the additional cropping seasons leading to an increase in sediment yield estimates for some agricultural HRUs. Nkwasa et al. (2022) recommended that global phenology datasets incorporate present-day multiple cropping seasons as these impact sediment yield estimates. Nevertheless, these results show that a realistic representation of the role of crop canopy cover in soil erosion and sediment yield modeling is essential.

The role of crop cover representation on sediment yield estimates in this study conforms to studies done by Abaci and Papanicolaou (2009) and Ma et al. (2019), which show how crop characteristics such as canopy or residue cover significantly amplify or deamplify the impact of precipitation on soil erosion in agricultural watersheds. Through interception of rain droplets, crop cover and crop residue left on the fields directly affect soil loss.

### 3.4 HRU adaptation

Within the 41 catchments selected from literature (Vanmaercke et al., 2014), the sediment yield peaked in the catchment size class of 20 – 40 km^2^ (Supporting material E; Fig. E1) consistent with the MUSLE range of application. Consequently, an area threshold of 40 km^2^ was selected for this region. Setting the threshold area to 40 km^2^ resulted into a 14.62 % reduction in the annual average regional sediment yield, with reductions varying from 0 % to 28 % in the HRUs (Fig. 4(c)). Using a 40 km^2^ area threshold assumes an HRU as a hydrologically isolated small catchment while staying in agreement with the size for which the original MUSLE was developed and tested. Additionally, the 40 km^2^ threshold area falls within the range recommended by Vigiak et al. (2015) from 0.01 km^2^ (accounting for hillslope process) up to 50 km^2^ (conceptually accounting for other sediment sources other than sheet and inter-rill erosion that dominates at the hillslope scale). The use of a threshold area above which the sediment yields are linearized is important in reducing sediment yields inflated by large HRU areas since the MUSLE can in part account for the increase in sediment yield but it cannot consider its decrease (Vigiak et al., 2015, Vigiak et al., 2017).

The decrease in the sediment yield estimates due to the HRU area threshold coupled with the decrease in the sediment yield estimates due to improved crop representation and an increase in sediment yield estimates due to slope adaptation shown in the previous sections, results to a combined 15.98 % increase in the annual average sediment yield estimates in comparison to the default model. This indicates that in some practical applications, the underestimation in the average sediment yield estimate due to the use of slope factors from a coarse DEM can be compensated for by an overestimation due to incorrect crop representation and large model units leading to partial error cancellation. However, this does not mean that the sediment yield simulation at the HRU level is spatially correct.

### 3.5 HMBC evaluation

**Fig. 5:**
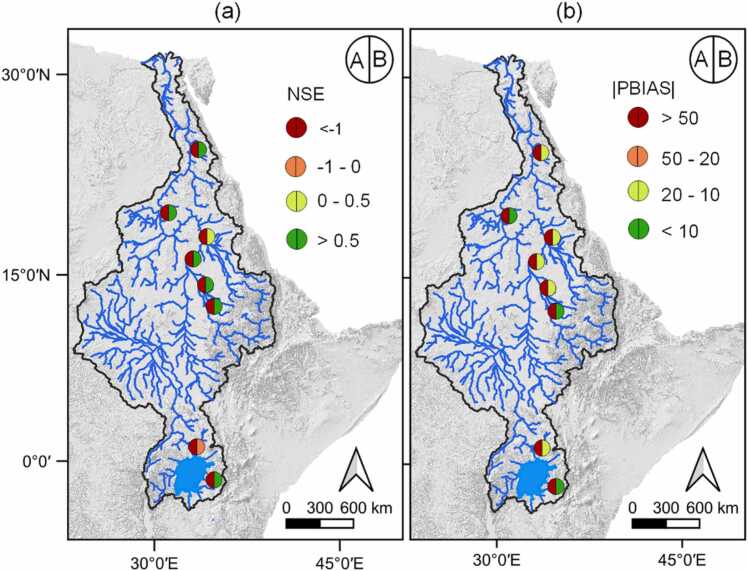
Model performance of NSE (a) and PBIAS (b) at flow gauging stations after implementing HMBC (A – default model and B – revised model performances).

The surface runoff ratio, ET ratio and groundwater ratio between the observed and simulated values improved towards the objective ratios derived from long-term data (Supporting Material B, Table B1), thus increasing the confidence in the representation of the water balance components. HMBC provides an opportunity for controlling the surface runoff generated within subbasins rather than just adjusting the flow results at the outlets or gauging stations. Fig. 5(a) and (b) show the statistical comparison of discharge at the GRDC gauging stations with 70 % of the stations having an NSE > 0.5, a significant improvement to the default model results. Most of the gauge stations with poor performance had extra abstractions from the corresponding rivers, which were not implemented in the model due to lack of detailed data. Degefu and He (2016) reported several unilateral water abstraction schemes within the Nile basin needed to meet the population's growing demand.

Since this study focuses on the land surface processes, a better representation of the water balance components, specifically, the surface runoff, improves the model's credibility in the estimation of HRU sediment yield using the MUSLE. The use of this approach with soft data makes it useful for data-scarce regions that would otherwise have not been calibrated.

### 3.6 Sediment yield spatial distribution

**Fig. 6:**
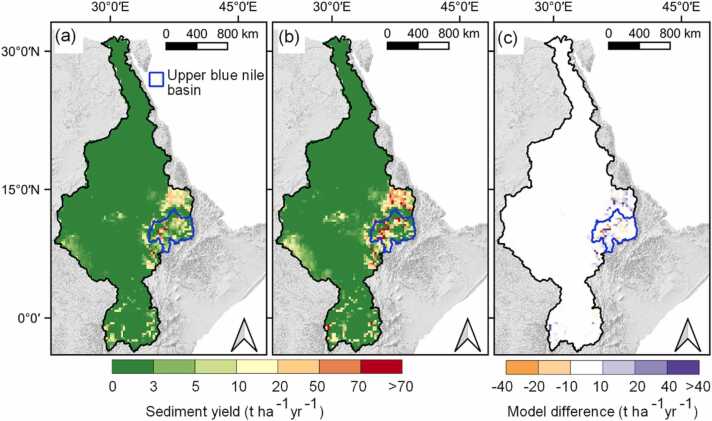
Simulated spatial annual average sediment yield in both the default (a) and revised (b) models; (c) model difference between the revised and default model sediment yield simulations (Revised simulation – Default simulation).

Fig. 6(a) and (b) show the spatial pattern of sediment yield estimates in both the default and revised model setups, respectively. A similar spatial trend is visible in both models with high sediment yield rates concentrated in the Ethiopian highlands, which is related to the region’s high slopes. However, the magnitude of sediment yield estimates is higher in the revised model than in the default model in most erosion-prone regions characterized by high sediment yield (Fig. 6(c)). The long term average annual sediment yield (1989 – 2019) estimated by the revised model is 1.79 t ha^−1^ yr^−1^, 27 % more than the default model estimate. Differences in sediment yield magnitudes are more noticeable in the Upper Blue Nile basin in Ethiopia, which has previously been reported to experience widespread soil erosion due to intensive cultivation, erosion-prone topography and climatic conditions (Belayneh et al., 2020).

The results of this study were first compared to previous studies that have either employed the same model (SWAT) in sediment yield estimates or reported sediment yield estimates within the region. From literature, the Upper Blue Nile basin is the most documented basin with SWAT applications within the Nile Basin region. The sediment yield estimates from the revised model in the Upper Blue Nile basin from this study ranged between 0 and 190 t ha^−1^ yr^−1^, which is comparable to the estimates from Betrie et al. (2011) that ranged from 0 to over 150 t ha^−1^ yr^−1^ while the sediment yield estimates from the default model were greatly underestimated (0–113 t ha^−1^ yr^−1^). In comparison to the Ethiopian national review of observed soil rates of 29.9 t ha^−1^ yr^−1^ in the Upper Blue Nile basin Haregeweyn et al. (2015b), the revised model estimates (21.7 t ha^−1^ yr^−1^) are comparable while the default model estimates (12.4 t ha^−1^ yr^−1^) are greatly underestimated. Hence, based on a plausibility check with previous SWAT studies in the Upper Blue basin, there is a consistent underestimation with the default model as the revised model gives estimates in comparable ranges. This underestimation is revealed in Fig. 6(c), which shows the difference between the revised and default models. Additional validation of the sediment yield estimates in Supporting material C, Table C1 shows a better performance of the revised model in comparison to reported estimates in the region.

#### 3.6.1 Comparison with measured catchment sediment yield

Considering only large catchments (area > 100 km^2^) from observed sediment yield data (Vanmaercke et al., 2014), a significant positive relationship exists (ρ < 5 %) for both the default and revised model estimates with the observed and reported sediment yield (Supporting material C, Fig. C1). However, the correlation ($r^{2}$) between the observed sediment yield and the simulated sediment yield estimates improved from 0.11 to 0.39 for the default and revised model simulations respectively. This improvement in the correlation provides insights on the credibility of the approaches made in the revised model towards improvement in the sediment yield estimates. This means that the revised SWAT+ sediment yield estimates from this study explain 39 % of the variance in observed sediment yield.

Using the same observed sediment yield data, Fenta et al. (2020) obtained an overall correlation, $r^{2}$ of 0.4 between RUSLE soil erosion estimates and sediment yield observations in East Africa. However, as noted by Vanmaercke et al. (2014), there is a strong bias in sediment yield observations towards erosion prone areas. Additionally, the sediment yield observations at the catchment outlet reflect several erosion processes such as gully erosion and river bank erosion which are not taken into account in this study. Hence, this relationship only considers rain-driven sheet erosion.

### 3.7 Evaluation of sediment load in the region

The long term annual average river sediment loads in the revised model were broadly comparable to the reported ranges of observations compared to the river sediment loads simulated by the default model. (Appendix A, Appendix A). This improvement in the sediment load estimates by the revised model can be attributed to both the methodological framework adaptations and the reservoir implementations. However, there was still a slight overestimation of sediment load at some gauging stations. Nevertheless, with a more detailed time series of measured river sediment loadings, the overestimation can be corrected through additional calibration of the peak channel velocities that plays a critical role in sediment transport by water (Lin et al., 2013).

Detailed calibration and validation would be carried out with better data availability in the region to make model predictions more accurate and improve the revised model's overall performance. However, the different plausibility checks and validation methods used in this study indicate that the revised SWAT+ model gives satisfactory sediment yield and sediment load estimates compared to the default model without performing detailed calibrations. This is especially of importance for data-scarce or large scale studies where detailed calibrations can not be conducted for practical reasons (lack of data or lack of computer power).

### 3.8 Sediment yield projections under climate change

**Fig. 7:**
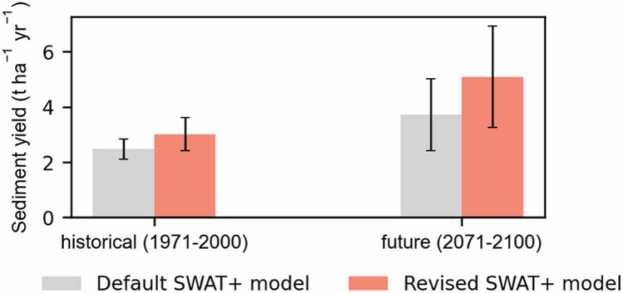
Mean annual sediment yield in the historical (1971 – 2000) and future (2071 – 2100) periods for the default and revised model setups.

A similar signal is found in both the default and revised model simulations, with annual average sediment yield increasing with climate change (Fig. 7). However, higher annual average sediment yield estimates with the revised model are observed in both the historical and future periods.

**Fig. 8:**
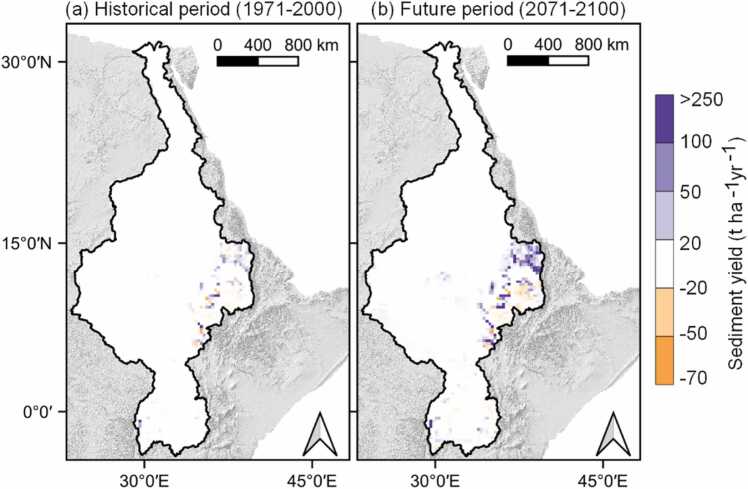
Spatial difference between the default and revised model setups calculated as; (Difference = Revised model – Default model) for the (a) historical period (1971 – 2000) and (b) future period (2071 – 2100).

The long term annual average sediment yield is projected to increase by 61.8 % (up to 5.2 ± 1.7 t ha^−1^ yr^−1^) in the revised model and by 39.1 % (up to 3.6 ± 1.3 t ha^−1^ yr^−1^) in the default model with future climate change. This difference shows that the proposed methodological adaptations during model set-up will affect the magnitude of results even if the climate signal remains the same. Fig. 8, further shows the spatial difference between the default and revised models in both the historical and future climate periods. As expected, the spatial difference is stronger during the future period due to the projected spatial increase in precipitation (Fig. 3), which increases the surface runoff, thereby increasing erositivity. However, the difference is more noticeable in the erosion-prone areas such as the Ethiopian highlands. Hence, the proposed methodological approaches have an influence if the model is to be used for sediment yield impact studies and, more especially, if the results are to be interpreted at the HRU (local) scale.

From the contemporary and future sediment yield estimates in the Nile basin, sediment yield is highest within the Ethiopian highlands which are identified as erosion hotspot areas. Previous studies (Vanmaercke et al., 2014) have considered sediment yield rates in Ethiopia as high by African standards. This has implications for not only Ethiopia but also downstream countries (Sudan and Egpyt) in the basin by increasing the sediment load in the Nile river with large quantities of eroded material. Downstream reservoirs e.g Merowe, GERD (Grand Renaissance Ethiopian Dam), Aswan are also on a threat of speedy sedimentation if the sediment yield rates continue. Thus, there is need for prioritization of soil erosion risks in the eastern part of the Nile basin which experiences high sediment yield, to support sustainable use of the basin’s land and water resources under both current and future climate change scenarios. In addition to climate change, land use practices and changes such as agricultural over-utilization, overgrazing and deforestation can easily facilitate soil erosion through loosening of the soil surface. Hence, future work is recommended targeting the impacts of current and future land use changes on sediment yield in the basin.

### 3.9 Limitations

In this study, we applied the SWAT+ model using the MUSLE at a regional scale to estimate sediment yield even though the MUSLE was developed for small catchments (< 40 km^2^) to predict sheet and rill erosion processes (Williams and Berndt, 1977). Subsequently, this model is not expected to perform well in areas where other erosion processes such as landslides and ephemeral erosion are dominant. Hence, modelers need to always be aware of the processes being modeled. The sensitivity approach used can be considered simplistic as the $K_{\mathrm{USLE}}$, $C_{\mathrm{USLE}}$, $P_{\mathrm{USLE}}$ and ${\mathrm{LS}}_{\mathrm{USLE}}$ factors easily represent linear scaling factors (Gwapedza et al., 2021). Future applications of the model could test the sensitivity of changes in HRU units on the erositivity factor. Another limitation to the modeling study is the sparse and poor quality records of river flow, reservoir data, plot-scale measurements and sediment loads which are considered high by regional standards (Conway, 2000; Haregeweyn et al., 2015a), making a proper validation of the model results challenging. With most of the sediment load in the region (ca. 90 %) flowing from the Ethiopian highlands, through the Blue Nile and Atbara rivers (Abdalla, 2013), most of the reported sediment load observations and estimations were for the Blue Nile basin. This limited a proper evaluation of the river sediment loads within the White Nile basin (lower part of the region). In addition, the scale of application in this study suggests that the required input data resolution for large scale process-based models like SWAT+ is currently lacking, and improvements are necessary for better performance of the models. For example, global phenology datasets with multiple cropping seasons are needed to better represent the agricultural practices that impact sediment yield estimation in the MUSLE.

## 4 Summary and conclusion

This paper demonstrates methodological approaches to improve SWAT+ sediment yield predictions and projections at regional scales under global change factors. An approach to overcome the sediment yield underestimations of a regional SWAT+ model due to topographic factors extracted from a coarse DEM has been proposed. This approach involves extracting topographic factors from a high/medium resolution DEM, resampling to the model resolution and replacing the topographic factors at the HRU level. We thus find that using this approach reduces the underestimations of sediment yield by using topographic factors extracted from a medium resolution DEM (90 m) than from a resampled DEM (250 m). However, if not bounded by computational constraints, using a higher DEM resolution throughout the model setup is recommended to minimize uncertainty in model simulations.

The incorporation of crop phenology and management practices significantly impacted sediment yield estimates, especially in the highly cultivated regions. Therefore, a better representation of crop phenology at the regional scale is recommended to minimize errors in sediment yield estimates since the SWAT+ model updates the cover management factor daily as the crop grows. Regarding the model unit size, introducing an area threshold (40 km^2^) above which the HRU sediment yields are linearized in relation to the HRU area helped overcome the risk of overestimating sediment yields in large HRUs. However, it is worth mentioning that the underestimation due to the coarse DEM and the over estimation due to the large HRU size coupled with incorrect crop representation can easily lead to partial error cancellation, which needs to be further investigated. For the hydrology component, although with limited availability of measured data, the HMBC approach adopted in this study leads to improved representation of processes in the model, specifically the surface runoff that is explicitly used in the MUSLE to estimate the volume of eroded material.

Overall, the sediment yield projections under the high-end climate change scenario (SSP5-RCP8.5) increase in both the default and revised model setups. However, there is a significant difference in sediment yield magnitude and spatial distribution between the two model setups. Thus, the methodological framework adopted in this study greatly influences the sediment yield projections estimates at HRU level. Consequently, to apply SWAT+ for regional sediment yield prediction and mapping, the following general protocol should be incorporated in the modeling framework:

(1) Use resampled topographic factors extracted from medium/fine resolution DEMs as opposed to topographic factors extracted from resampled DEMs, unless restricted by computational capacity.
(2) Adopt an area threshold in the MUSLE above which sediment yields are linearized in relation to the HRU area to avoid overestimating the HRU sediment yield.
(3) Incorporate crop phenology and agricultural management in regional SWAT+ model applications to improve crop cover estimation.
(4) Apply the HMBC to improve the hydrologic component of the model with a specific interest in the surface runoff prediction.

## Software

The SWAT+ model is publicly available at https://swat.tamu.edu/software/plus/. Analysis and processing of data was done using Python and R scripts. Scripts can be obtained from the corresponding author upon request.

## CRediT authorship contribution statement

**Albert Nkwasa:** Conceptualization. **Ann van Griensven**: Conceptualization. **Albert Nkwasa:** Methodology, Investigation, Analysis. **Celray James Chawanda**: Methodology, Investigation, Analysis. **All authors**: Result interpretation, Writing – review & editing. **Ann van Griensven**: Funding acquisition.

## Declaration of Competing Interest

The authors declare that they have no known competing financial interests or personal relationships that could have appeared to influence the work reported in this paper.
