# Supplementary material for: Regionalization of the SWAT+ model for projecting climate change impacts on sediment yield: An application in the Nile basin
Source: J Hydrol Reg Stud. 2022 Aug;42:101152. doi: 10.1016/j.ejrh.2022.101152 (PMC9350554; doi:10.1016/j.ejrh.2022.101152)
Supplement: Supplementary file 1 — Supplementary material [file mmc1.zip › supporting_material_EJRH_EJRH-D-22-00264/Supporting material A.docx]

**Journal name:** Journal of Hydrology - Regional Studies

*Supporting material of.*

**Regionalization of the SWAT+ model for projecting climate change impacts on sediment yield: An application in the Nile basin**

Albert Nkwasa et al.

Correspondence to: Albert Nkwasa (albert.nkwasa@vub.be)

**Supporting material, A: Topography factor adaptation**

To understand the effect of topographic factors extracted from fine/medium DEM resolution and factors extracted from a coarse DEM resolution on the SWAT+ sediment yield estimates, the annual average sediment yield output from different test model setups in the Blue Nile basin (within the Nile basin), with different topographic factors was compared in Table A1. The Blue Nile model tests were uncalibrated but only checked for the water balance. This approach isolated the uncertainty in the model due to different topographic factors.

Using the model at 90 m DEM resolution as the reference, it was observed that the sediment yield estimates reduced with reducing DEM resolution in Table A1. The decrease of the sediment yield estimates was more notable in model setups with topographic parameters from resampled DEMs, than in model setups using resampled topographic parameters extracted from a medium resolution DEM (90 m). There was a significant reduction in the error difference (difference between model setups and the reference 90 m DEM model) with model setups utilizing resampled topographic parameters from a medium resolution DEM.

Table A1: Annual sediment yield estimates using (a) Topographic parameters from resampled DEM, (b) Resampled topographic parameters from medium resolution DEM (90 m)

| Model DEM resolution | Annual average sediment yield (t/ha/yr) | |  | Error difference (%) | |
| --- | --- | --- | --- | --- | --- |
|  | (a) | (b) |  | (a) | (b) |
| 90 m | 13.75 | - |  | - | - |
| 250 m | 10.97 | 12.78 |  | 20.2 | 6.9 |
| 500 m | 8.61 | 11.62 |  | 37.4 | 15.2 |
| 100 m | 6.88 | 9.68 |  | 50.1 | 29.6 |

This result conforms to the study by Grohmann (2015) that recommended the use of high/medium resolution data to derive basic topographic parameters, and resample to a coarser resolution as needed.

**References**

Grohmann, C.H., 2015. Effects of spatial resolution on slope and aspect derivation for regional-scale analysis. Comput. Geosci. 77, 111–117. https://doi.org/10.1016/j.cageo.2015.02.003
