# Supplementary material for: Regionalization of the SWAT+ model for projecting climate change impacts on sediment yield: An application in the Nile basin
Source: J Hydrol Reg Stud. 2022 Aug;42:101152. doi: 10.1016/j.ejrh.2022.101152 (PMC9350554; doi:10.1016/j.ejrh.2022.101152)
Supplement: Supplementary file 1 — Supplementary material [file mmc1.zip › supporting_material_EJRH_EJRH-D-22-00264/Supporting material B.docx]

**Journal name:** Journal of Hydrology - Regional Studies

*Supporting material of.*

**Regionalization of the SWAT+ model for projecting climate change impacts on sediment yield: An application in the Nile basin**

Albert Nkwasa et al.

Correspondence to: Albert Nkwasa (albert.nkwasa@vub.be)

**Supporting material, B: Hydrologic Mass Balance Calibration (HMBC)**


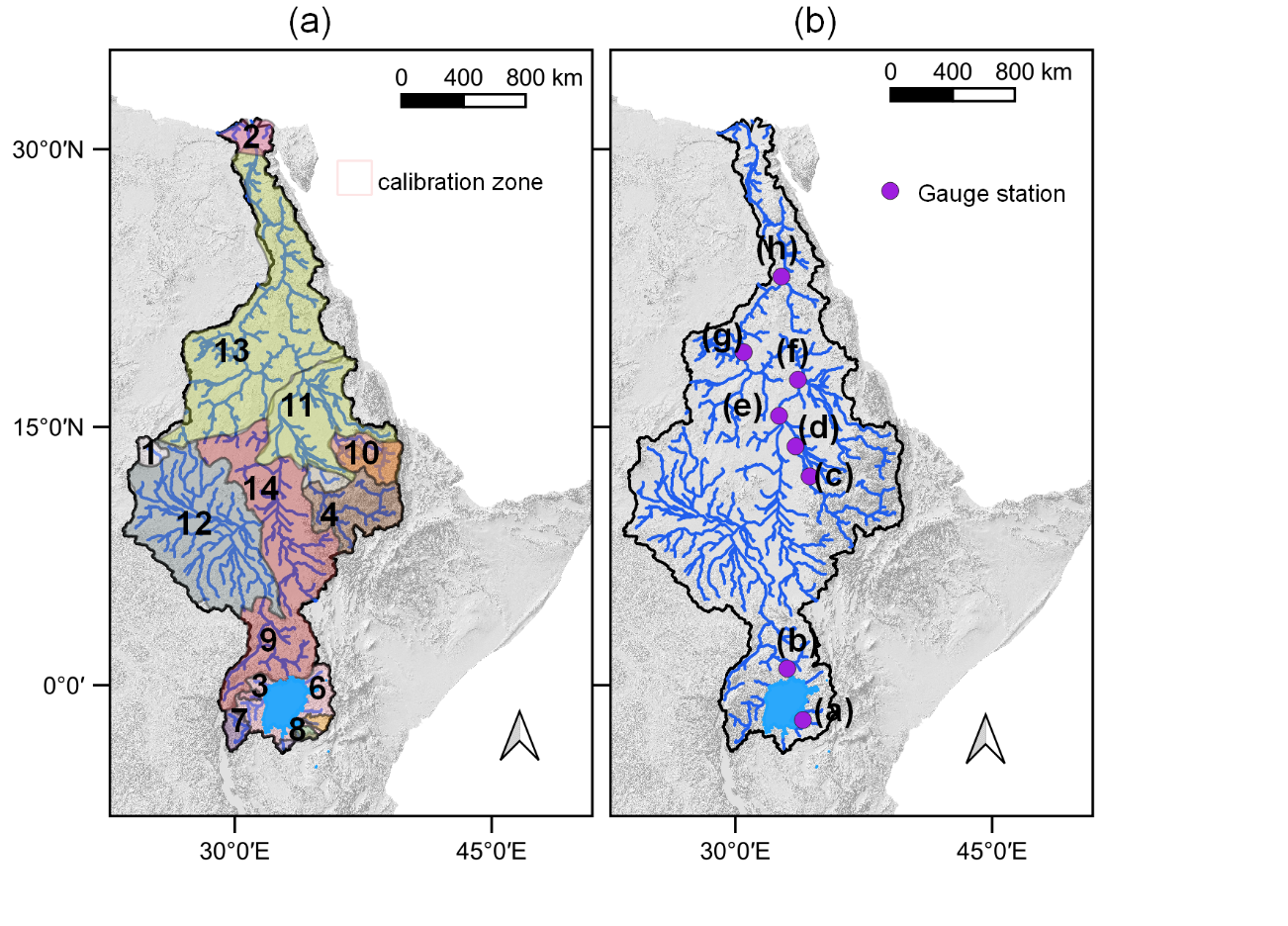


Figure B1: (a) Division of the study area into 14 calibration zones; (b) Location of the gauging stations

Table B1: Mass balance calibration ratios for zones (Figure B1 (a)) in the Nile basin (sr = surface runoff ratio, et = ET ratio, gw = ground water ratio)

|  | Objective | | |  | Default | | |  | Calibrated | | |
| --- | --- | --- | --- | --- | --- | --- | --- | --- | --- | --- | --- |
| region | sr | et | gw |  | sr | et | gw |  | sr | et | gw |
| 1 | 0.128 | 0.716 | 0.156 |  | 0.224 | 0.692 | 0.084 |  | 0.129 | 0.713 | 0.158 |
| 2 | - | 0.704 | 0.296 |  | 0.051 | 0.892 | 0.057 |  | 0.059 | 0.823 | 0.117 |
| 3 | 0.034 | 0.913 | 0.052 |  | 0.097 | 0.866 | 0.038 |  | 0.045 | 0.892 | 0.062 |
| 4 | 0.096 | 0.835 | 0.069 |  | 0.045 | 0.907 | 0.048 |  | 0.091 | 0.849 | 0.060 |
| 5 | 0.061 | 0.709 | 0.230 |  | 0.017 | 0.889 | 0.095 |  | 0.081 | 0.748 | 0.171 |
| 6 | 0.091 | 0.860 | 0.049 |  | 0.004 | 0.689 | 0.307 |  | 0.126 | 0.826 | 0.048 |
| 7 | - | 0.873 | 0.127 |  | 0.003 | 0.721 | 0.276 |  | 0.008 | 0.813 | 0.179 |
| 8 | 0.120 | 0.751 | 0.129 |  | 0.148 | 0.831 | 0.021 |  | 0.124 | 0.766 | 0.110 |
| 9 | 0.093 | 0.731 | 0.176 |  | 0.083 | 0.849 | 0.068 |  | 0.090 | 0.796 | 0.113 |
| 10 | 0.019 | 0.901 | 0.080 |  | 0.147 | 0.798 | 0.056 |  | 0.072 | 0.871 | 0.057 |
| 11 | 0.022 | 0.758 | 0.219 |  | 0.033 | 0.955 | 0.012 |  | 0.024 | 0.808 | 0.168 |
| 12 | 0.066 | 0.880 | 0.054 |  | 0.034 | 0.950 | 0.017 |  | 0.063 | 0.881 | 0.056 |
| 13 | 0.012 | 0.897 | 0.091 |  | 0.038 | 0.936 | 0.026 |  | 0.014 | 0.902 | 0.084 |
| 14 | 0.017 | 0.841 | 0.142 |  | 0.031 | 0.952 | 0.016 |  | 0.015 | 0.869 | 0.116 |


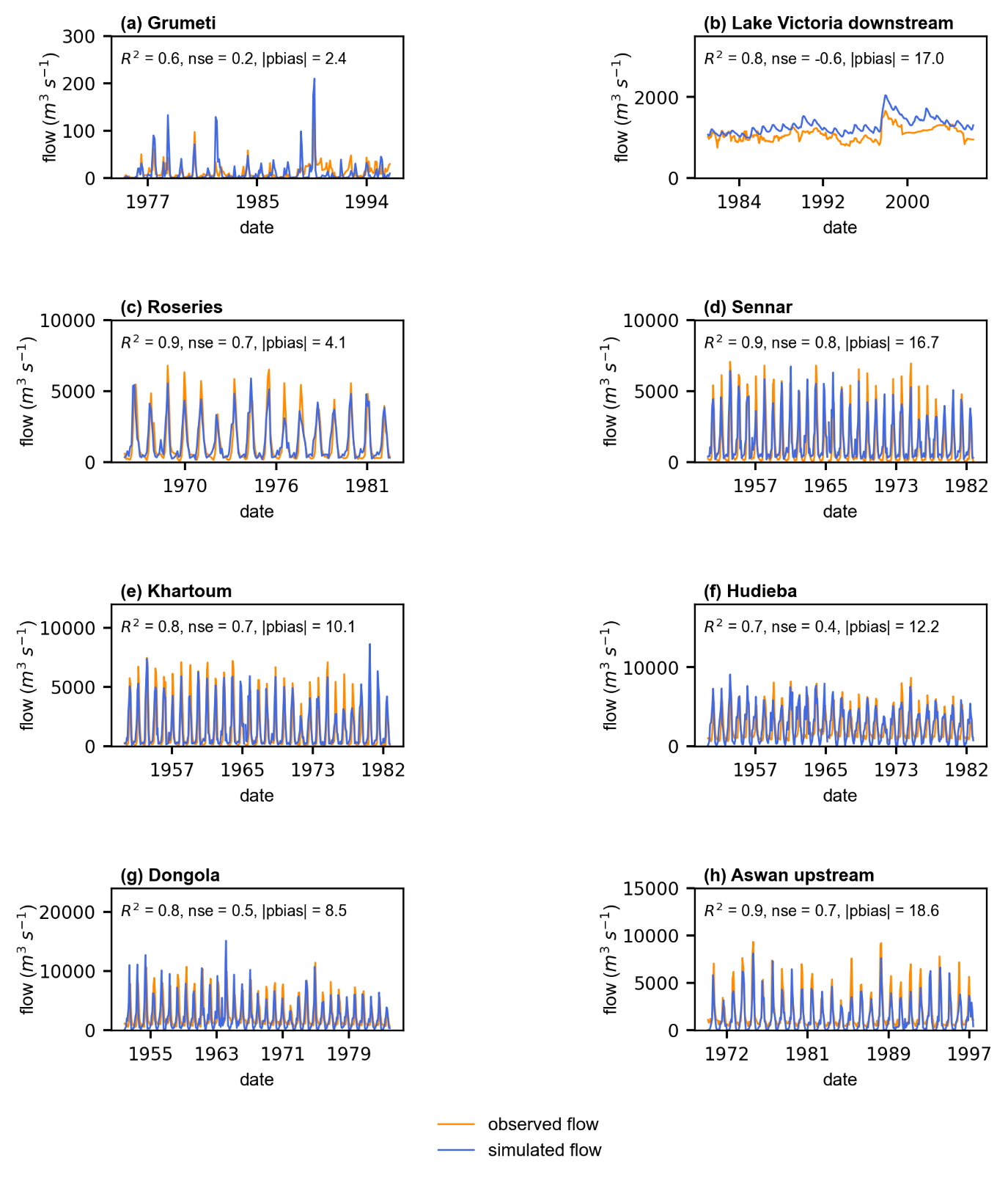


Figure B2: Simulated and observed flow at selected gauging stations (Figure B1 (b)) in the basin

Table B2: Revised model performance indicators

| Station | Performance indicators | |
| --- | --- | --- |
|  | NSE | \|PBIAS\| |
| Aswan upstream | 0.7 | 18.6 |
| Dongola | 0.5 | 8.5 |
| Hudieba | 0.4 | 12.2 |
| Khartoum | 0.7 | 10.1 |
| Sennar | 0.8 | 16.7 |
| Roseries | 0.7 | 4.1 |
| Lake Victoria downstream | -0.6 | 17.0 |
| Grumeti | 0.2 | 2.4 |
