# Supplementary material for: Regionalization of the SWAT+ model for projecting climate change impacts on sediment yield: An application in the Nile basin
Source: J Hydrol Reg Stud. 2022 Aug;42:101152. doi: 10.1016/j.ejrh.2022.101152 (PMC9350554; doi:10.1016/j.ejrh.2022.101152)
Supplement: Supplementary file 1 — Supplementary material [file mmc1.zip › supporting_material_EJRH_EJRH-D-22-00264/Supporting material C.docx]

**Journal name:** Journal of Hydrology - Regional Studies

*Supporting material of.*

**Regionalization of the SWAT+ model for projecting climate change impacts on sediment yield: An application in the Nile basin**

Albert Nkwasa et al.

Correspondence to: Albert Nkwasa (albert.nkwasa@vub.be)

**Supporting material, C: Sediment yield validation**

Table C1: Comparison of simulated sediment yield and reported sediment yield estimates

| Default model (t ha^-1^ yr^-1^) | Revised model (t ha^-1^ yr^-1^) | Reported/observed (t ha^-1^ yr^-1^) | Reference | Location |
| --- | --- | --- | --- | --- |
| 1 – 113 | 1 – 190 | 1 - >150 | (Betrie et al., 2011) | Upper Blue Nile catchment |
| 16 | 41 | 50 - 80 | (Lemma et al., 2019) | Rib catchment |
| 5.9 | 8.3 | 10.5 | (Haregeweyn et al., 2005) | Tigary catchment |
| 13.7 | 14.8 | 15.2 | (Welde, 2016) | Tekeza catchment |
| 11.1 | 19.9 | 16.2 | (Asres and Awulachew, 2010) | Gumara catchment |


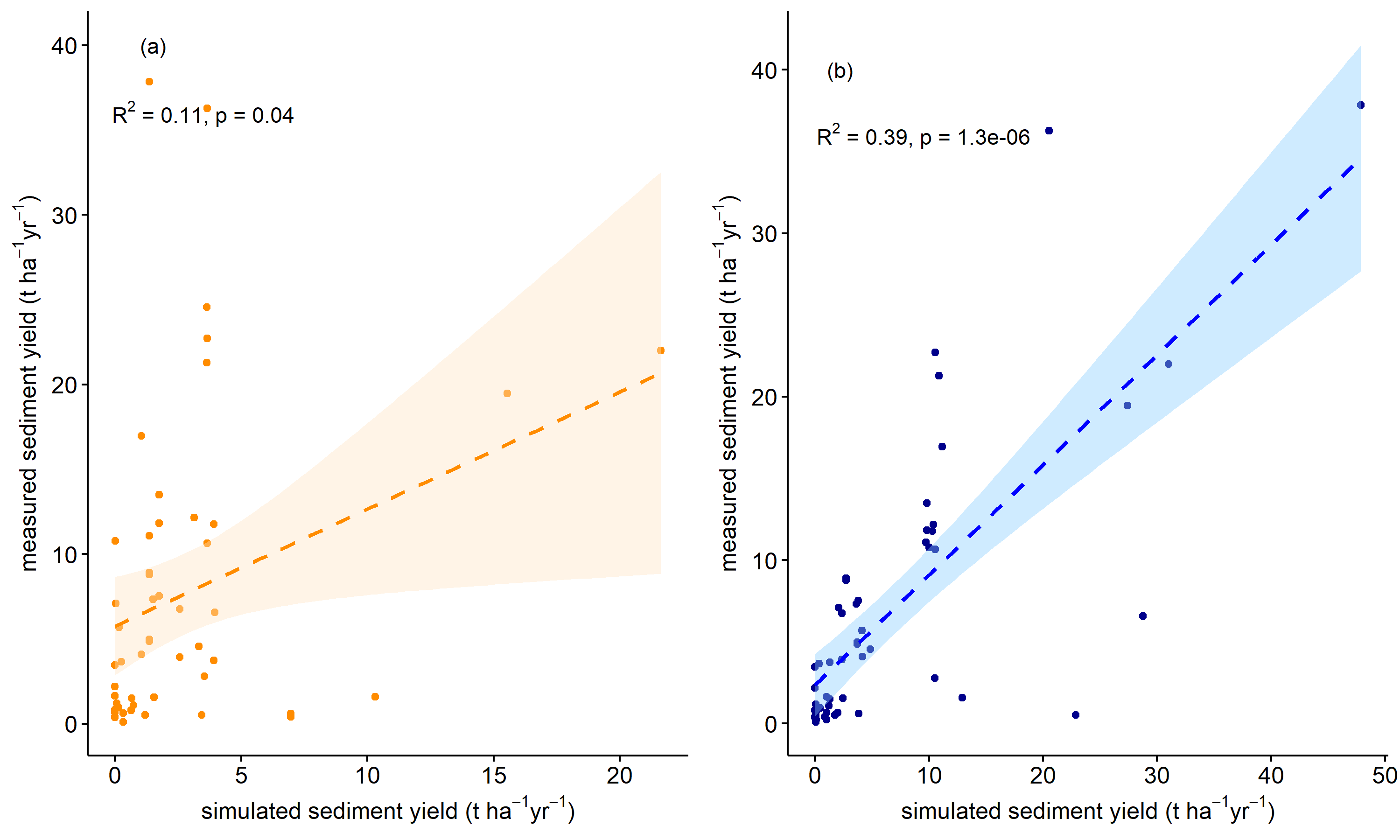


Figure C1: (a) Default model sediment yield estimates versus observed catchment sediment yield (Vanmaercke et al., 2014); (b) Revised model sediment yield estimates versus observed catchment sediment yield

**Reference**

Asres, M.T., Awulachew, S.B., 2010. SWAT based runoff and sediment yield modelling: a case study of the Gumera watershed in the Blue Nile basin. Ecohydrol. Hydrobiol., Invited contributions from the International Symposium Ecohydrology for water ecosystems and society in Ethiopia Addis Ababa, Ethiopia, 18-20 November 2009 10, 191–199. https://doi.org/10.2478/v10104-011-0020-9

Betrie, G.D., Mohamed, Y.A., van Griensven, A., Srinivasan, R., 2011. Sediment management modelling in the Blue Nile Basin using SWAT model. Hydrol. Earth Syst. Sci. 15, 807–818. https://doi.org/10.5194/hess-15-807-2011

Haregeweyn, N., Poesen, J., Nyssen, J., Verstraeten, G., de Vente, J., Govers, G., Deckers, S., Moeyersons, J., 2005. Specific sediment yield in Tigray-Northern Ethiopia: Assessment and semi-quantitative modelling. Geomorphology 69, 315–331. https://doi.org/10.1016/j.geomorph.2005.02.001

Lemma, H., Frankl, A., van Griensven, A., Poesen, J., Adgo, E., Nyssen, J., 2019. Identifying erosion hotspots in Lake Tana Basin from a multisite Soil and Water Assessment Tool validation: Opportunity for land managers. Land Degrad. Dev. 30, 1449–1467. https://doi.org/10.1002/ldr.3332

Vanmaercke, M., Poesen, J., Broeckx, J., Nyssen, J., 2014. Sediment yield in Africa. Earth-Sci. Rev. 136, 350–368. https://doi.org/10.1016/j.earscirev.2014.06.004

Welde, K., 2016. Identification and prioritization of subwatersheds for land and water management in Tekeze dam watershed, Northern Ethiopia. Int. Soil Water Conserv. Res. 4, 30–38. https://doi.org/10.1016/j.iswcr.2016.02.006
