# Supplementary material for: Regionalization of the SWAT+ model for projecting climate change impacts on sediment yield: An application in the Nile basin
Source: J Hydrol Reg Stud. 2022 Aug;42:101152. doi: 10.1016/j.ejrh.2022.101152 (PMC9350554; doi:10.1016/j.ejrh.2022.101152)
Supplement: Supplementary file 1 — Supplementary material [file mmc1.zip › supporting_material_EJRH_EJRH-D-22-00264/Supporting material D.docx]

**Journal name:** Journal of Hydrology - Regional Studies

*Supporting material of.*

**Regionalization of the SWAT+ model for projecting climate change impacts on sediment yield: An application in the Nile basin**

Albert Nkwasa et al.

Correspondence to: Albert Nkwasa (albert.nkwasa@vub.be)

**Supporting material, D: Crop cover simulation**

Figure D1 (a) shows the spatial distribution of the agricultural area within the Nile basin. To illustrate the impact of the improved crop representation, the Upper Blue Nile catchment was selected. LAI plots of rainfed wheat (Figure D1 (b)) and rainfed maize (Figure D1 (d)) show an improved temporal pattern of LAI that correlates with the rainfall season in the revised SWAT+ model as compared to the default SWAT+ model. Subsequently, this impacts the crop cover factor estimation (Figure D1 (c) and Figure D1 (e)) as the crop cover factor is lowest in the wet season as simulated by the revised SWAT+ model. Hence, signifying a very high crop cover protecting the topsoil against soil erosion (Schönbrodt et al., 2010). This results into reduced sediment yield estimates in cultivated areas in the revised SWAT+ model with improved crop cover representation as compared to the default SWAT+ model.


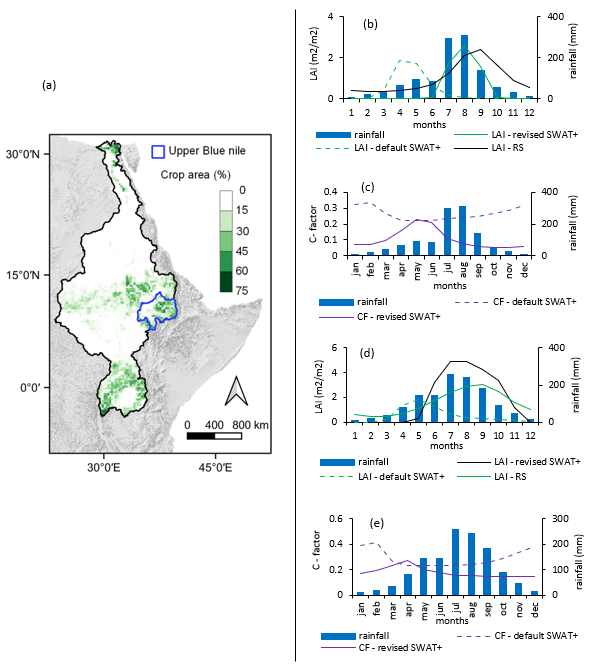


Figure D1: (a) crop area in the region; (b) LAI comparison for rainfed wheat, (c) Crop Cover Factor (CF) comparison for rainfed wheat (d) LAI comparison for rainfed maize, (e) Crop Cover Factor (CF) comparison for rainfed maize in the Upper Blue Nile basin for selected HRUs.

**Reference**

Schönbrodt, S., Saumer, P., Behrens, T., Seeber, C., Scholten, T., 2010. Assessing the USLE crop and management factor C for soil erosion modeling in a large mountainous watershed in Central China. J. Earth Sci. 21, 835–845. https://doi.org/10.1007/s12583-010-0135-8
