# Supplementary material for: Regionalization of the SWAT+ model for projecting climate change impacts on sediment yield: An application in the Nile basin
Source: J Hydrol Reg Stud. 2022 Aug;42:101152. doi: 10.1016/j.ejrh.2022.101152 (PMC9350554; doi:10.1016/j.ejrh.2022.101152)
Supplement: Supplementary file 1 — Supplementary material [file mmc1.zip › supporting_material_EJRH_EJRH-D-22-00264/Supporting material E.docx]

**Journal name:** Journal of Hydrology - Regional Studies

*Supporting material of.*

**Regionalization of the SWAT+ model for projecting climate change impacts on sediment yield: An application in the Nile basin**

Albert Nkwasa et al.

Correspondence to: Albert Nkwasa (albert.nkwasa@vub.be)

**Supporting material, E: HRU area threshold selection**


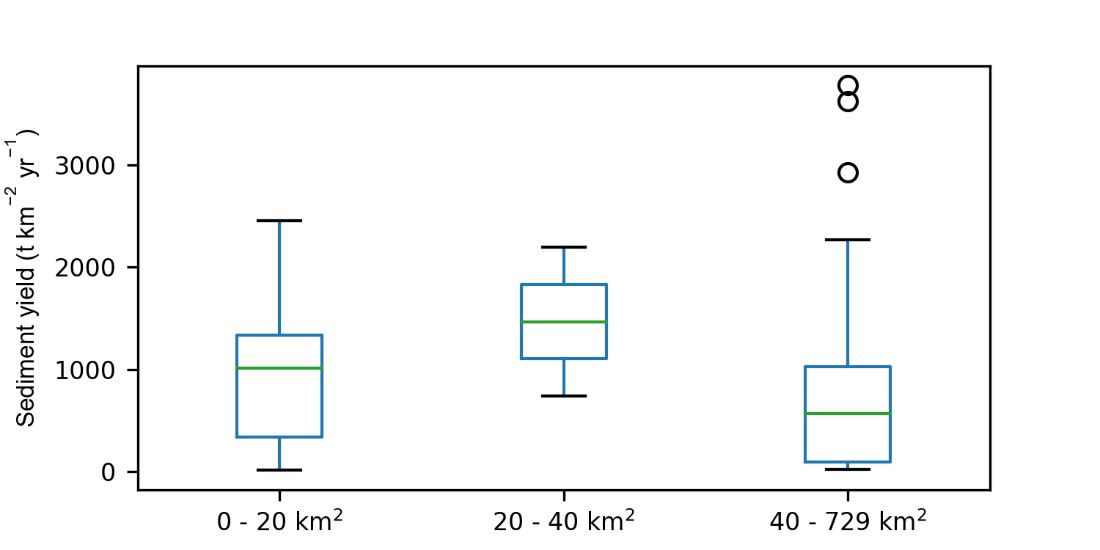


Figure E1: Distribution of average sediment yield observed in 41 catchments of size up to 625 km2 in the Nile basin used to select the MUSLE threshold area, ($A_{t}$)
