# Supplementary material for: Regionalization of the SWAT+ model for projecting climate change impacts on sediment yield: An application in the Nile basin
Source: J Hydrol Reg Stud. 2022 Aug;42:101152. doi: 10.1016/j.ejrh.2022.101152 (PMC9350554; doi:10.1016/j.ejrh.2022.101152)
Supplement: Supplementary file 1 — Supplementary material [file mmc1.zip › supporting_material_EJRH_EJRH-D-22-00264/Supporting material F.docx]

**Journal name:** Journal of Hydrology - Regional Studies

*Supporting material of.*

**Regionalization of the SWAT+ model for projecting climate change impacts on sediment yield: An application in the Nile basin**

Albert Nkwasa et al.

Correspondence to: Albert Nkwasa (albert.nkwasa@vub.be)

**Supporting material, F: Sediment load validation**


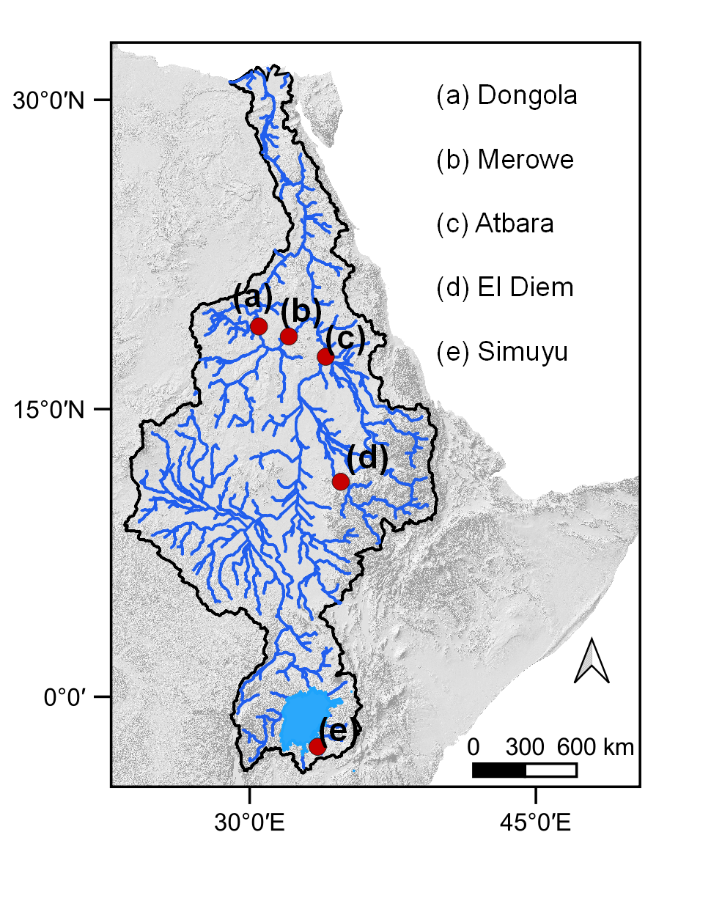


Figure F1: Location of measured and reported sediment river load gauging stations

Table F1: Comparison of simulated sediment load and measured or reported sediment load from literature

| Gauging station | River | Sediment load (million t yr^-1^) | | | Source |
| --- | --- | --- | --- | --- | --- |
|  |  | Measured/ reported | Default model | Revised model |  |
| Dongola | Nile | 140 - 160 | 178 | 166 | (El Monshid et al., 1997; Shalash, 1980) |
| El-diem | Blue Nile | 118 - 170 | 131 | 158 | (Ali et al., 2014) |
| Merowe | Nile | 137 - 180 | 107 | 128 | (Eizel-Din et al., 2010; Kantoush and Sumi, 2013) |
| Atbara | Atbara | 30 - 82 | 74.3 | 89 | (Zenebe, 2009; Garzanti et al., 2006; Williams, 2012) |
| Simuyu | Simuyu | 1.9 | 0.9 | 1.3 | (Zhang et al., 2020) |

**References**

Ali, Y.S.A., Crosato, A., Mohamed, Y.A., Abdalla, S.H., Wright, N.G., 2014. Sediment balances in the Blue Nile River Basin. Int. J. Sediment Res. 29, 316–328. https://doi.org/10.1016/S1001-6279(14)60047-0

Eizel-Din, M.A., Bui, M.-D., Rutschmann, P., Failer, E., Grass, C., Kramer, K., Hussein, A.S., Saghayroon-Elzein, A., 2010. Trap efficiency of reservoirs on the Nile River. River Flow 2010 1111–1118.

El Monshid, B.E.F., El Awad, O.M.A., Ahmed, S.E., 1997. Environmental effect of the Blue Nile sediment on reservoirs and irrigation canals, in: International 5th Nile 2002 Conference, Addis Ababa, Ethiopia.

Garzanti, E., Andò, S., Vezzoli, G., Megid, A.A.A., El Kammar, A., 2006. Petrology of Nile River sands (Ethiopia and Sudan): sediment budgets and erosion patterns. Earth Planet. Sci. Lett. 252, 327–341.

Kantoush, S.A., Sumi, T., 2013. Reservoir sedimentation and sediment management techniques in the Nile River basin countries, in: 12th International Symposium on River Sedimentation, Kyoto.

Shalash, S., 1980. Effect of sedimentation on storage capacity of high Aswan Dam Lake. Nile Res. Inst. NRI Qanater.

Williams, M., 2012. River sediments. Philos. Trans. R. Soc. Math. Phys. Eng. Sci. 370, 2093–2122. https://doi.org/10.1098/rsta.2011.0504

Zenebe, A., 2009. Assessment of spatial and temporal variability of river discharge, sediment yield and sediment-fixed nutrient export in Geba River catchment, northern Ethiopia. Leuven Dep. Earth Environ. Sci. KU Leuven.

Zhang, G., Majaliwa, M.J., Xie, J., 2020. Leveraging the Landscape: Case Study of Erosion Control through Land Management in the Lake Victoria Basin.
